# Power spectrum: A detailed dataset on electric demand and environmental interplays

**DOI:** 10.1016/j.dib.2023.109788

**Published:** 2023-12-06

**Authors:** M.S. Jawad, Chitra Dhawale, Abdel Rahman Al Ali, Azizul Azhar Bin Ramli

**Affiliations:** Datta Meghe Institute of Innovation and Research, FSKTM Faculty, Uthm University, Malaysia

**Keywords:** Electricity demand forecasting, Environmental parameters, Electrical load metrics, Data acquisition

## Abstract

This dataset provides detailed electricity demand forecasting metrics for the Sharjah Electricity and Water Authority (SEWA) over 2020 and 2021. Data encompasses both hourly and daily demand patterns, enriched with specific environmental parameters such as temperature, humidity, and solar irradiance. Additionally, SEWA's unique load metrics and lagged demand values, representing previous hour demand, are included.

Data was procured using advanced electrical load meters and standardized weather data acquisition systems. Preliminary and advanced data processing was conducted via Excel tool. This comprehensive dataset is invaluable for stakeholders in electricity provisioning and policy-making. Its granular detail makes it a pivotal resource for modelling and forecasting electricity demand, aiding in infrastructure planning, renewable energy considerations, and demand-side management. The potential applications span across academic, policy, and industry domains, rendering it a versatile tool for future electricity demand research.

Specifications Table

**Table:** 

Subject	Energy and Environmental Sciences
Specific subject area	Electricity demand forecasting considering environmental factors and load metrics.
Data format	Raw, Analysed, Filtered
Type of data	Table, Figure
Data collection	The dataset combines electricity demand, weather data, and SEWA load metrics. For electricity demand, advanced smart meters were employed across the grid to capture hourly and daily metrics. Daily weather data was sourced from meteorological stations, detailing various climatic conditions affecting electricity demand. SEWA load metrics were derived directly from SEWA's own monitoring and recording infrastructure. Data normalization was carried out using standard z-score methods to account for outliers and to ensure a uniform scale across various data points.
Data source location	Sharja Electricity, Water and Gas Authority (SEWA)—United Arab Emirates (UAE)
Data Availability	**Repository name:** Mendeley Data **Data identification number:**10.17632/4rjc87zrd3.2 Direct URL to data: SEWA Electricity Deman Forecasting – 2020 and 2021 - Mendeley Data **(**Published: 11 October 2023|Version 2|DOI:10.17632/4rjc87zrd3.2**)**
Data Accessibility	**Instructions for accessing these data:** **Access the Mendeley Portal** Open your preferred web browser. Enter the URL: SEWA Electricity Deman Forecasting – 2020 and 2021 - Mendeley Data into the address bar and hit enter. **Locate the Dataset** Once the page loads, you should be directed to the draft page for the "SEWA Electricity Demand Forecasting 2020-2021" dataset. Ensure that the dataset's title and other details match what you're looking for. **Data Download** Scan the page for a 'Download' button or section, typically located next to the dataset files or at the top of the page. If there are multiple files, you might see an option to 'Download All' or to download individual files. Click on the 'Download' or 'Download All' button. The dataset files should start downloading to your default downloads directory. **Viewing Dataset Documentation** Mendeley Data often provides dataset descriptions, methodologies, and other relevant information directly on the dataset page. Scroll through the dataset page to read any accompanying documentation or metadata that provides insight into the dataset, its variables, and collection methods. **Decompression (if needed)** If the dataset downloads as a compressed file (e.g., .zip format), use suitable software like WinRAR or 7-zip, or use built-in operating system features to extract the data files. **Data Analysis and Software Compatibility** Once downloaded, the dataset can be viewed and analysed using software or tools compatible with the file format (e.g., Microsoft Excel for .csv or .xlsx files). For specialized data formats, ensure you have the appropriate software or tools installed.

## 1 Value of the Data

• Longitudinal Insight: Offers insights over two years (2020 and 2021) for studying trends and changes over time.
• COVID-19 Impact: Reflects the impact of COVID-19 lockdowns on electricity demand, providing data for understanding external factors' effects on energy consumption.
• Seasonal Variability: Enables the examination of seasonal variations in electricity load and weather conditions.
• Geographic Focus: Pertains to the Emirate of Sharjah, UAE, relevant for regional energy dynamics analysis.
• Multi-Dimensional: Includes diverse measurements (e.g., temperature, humidity, load) for a comprehensive view of environmental and energy factors.
• Comparative Analysis: Facilitates comparisons between 2020 pandemic-related data and 2021 post-pandemic data.
• Research Applications: Useful for energy management, climate studies, and policy development, contributing to sustainability insights.

## 2 Data Description

These datasets are geographically specific, related to the Emirate of Sharjah, UAE. They are valuable for understanding regional energy dynamics, making them particularly relevant for local policymakers and researchers. The SEWA electricity forecasting dataset is a comprehensive collection of data spanning the years 2020 and 2021, meticulously tailored to assist in detailed power demand analysis. The dataset incorporates general daily parameters such as dates and specific environmental conditions. Hourly specifics, including attributes like temperature, humidity, wind speed, and solar irradiance, enrich the dataset, ensuring the provision of vital context to the demand metrics. Beyond the general parameters, the dataset pivots towards the more technical metrics linked with SEWA's electrical load dynamics. Aspects like the minimal electrical load, peak load, and energy per hour serve as the pivotal core of the collection. Additionally, the dataset introduces a dynamic perspective by integrating the “lagged demand” feature, representing the electricity demand from the preceding hour.

Structurally, the dataset is adeptly organized into hourly and daily breakdowns. The hourly data dissects power electricity demand for each hour of the day, from 0:00 to 24:00, while the daily representation amalgamates this information, presenting each day's cumulative demand.

From a statistical standpoint, this dataset is designed for an in-depth analysis. Researchers can compute average demand, identify variability in hourly demand, distinguish between peak and off-peak hours, and discern evolving trends over days and periods. Visually, it provides the scope for discerning daily demand patterns and understanding the evolution of demand over time. Moreover, the dataset empowers analysts to unearth deeper insights, unveiling seasonal fluctuations, long-term trends, and any cyclical or anomalous patterns inherent in the data. For those inclined towards advanced forecasting, the dataset is ripe for applications of models like ARIMA, Holt-Winters, or LSTM. Decomposition into trend, seasonal, and residual components can be executed, and monthly demand prediction becomes plausible when aggregating data.

Upon reviewing the “2020 Hourly Collected Data Sheet,” it does indeed appear that there are eight different measurements grouped together, with their sum shown in the ninth row. This grouping and summation of data may have been done to simplify the presentation of the information and provide an aggregate view of the eight measurements. This approach can make it easier to interpret the data.

The 2021 hourly data, on the other hand, are presented differently from the 2020 data. In the 2021 dataset, each measurement is provided individually for every hour without being grouped and summed in the same manner as the 2020 data. The difference in presentation could be due to various factors, including changes in data collection and reporting practices, or it may reflect different data requirements or analysis needs for the two years. Overall, the choice to group and sum certain measurements in the 2020 dataset and present the 2021 data differently could be driven by specific data management or analytical considerations, making it easier to work with and analyse the data for the respective years.

In essence, the SEWA electricity forecasting dataset is a holistic ensemble of data and metrics, carefully curated to offer researchers and analysts a thorough framework for electricity demand analysis and forecasting (Table 1).

**Table 1:** Collected data and analysis specifications of electricity demand forecasting data set.

Aspect	Factor under Analysis	Details/Insights
Data Structure	Hourly data breakdown.	Hourly power electricity demand from 0:00 to 24:00.
	Daily data representation.	Each row represents demand values for a day (or period).
Descriptive Statistics	Average demand calculation.	Insight into daily demand variation.
	Hourly demand variability.	Compute standard deviation for hourly variability.
	Peak vs. off-peak analysis.	Identify peak and off-peak hours based on consistent demand.
	Trend over days/periods.	Explore trends over days/periods.
Patterns	Seasonal fluctuations.	Explore seasonality in daily data.
	Long-term trends.	Identify any upward/downward trends.
	Cyclical or anomalous patterns.	Examine for cyclical patterns or anomalies.
Advanced Analysis	Forecasting future demand.	Consider forecasting models like ARIMA, Holt-Winters, or LSTM.
	Decomposition of time series.	Decompose into trend, seasonal, and residual components.
	Monthly demand prediction.	Aggregate data for monthly totals and forecast using suitable models, considering factors like previous months, yearly trends, and possible events affecting demand.

### 2.1 Repository structure

Folder: Electricity Demand Data 2020-2021

**Fig. 1:**
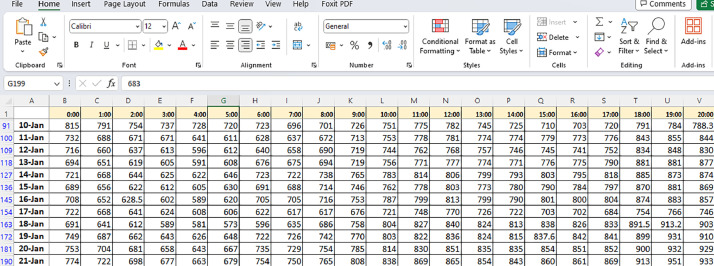
2020 hourly collected data for electricity demand.

Excel sheet-1: Hourly_Demand_2020.xlsx: This file provides a comprehensive record of hourly electricity demand for the entire year of 2020. Each row indicates a specific day, with columns breaking down the demand for each hour from 0:00 to 24:00. The following Fig. 1 depicts the hourly collected data for electricity demand in each month of the year of 2020.

**Fig. 2:**
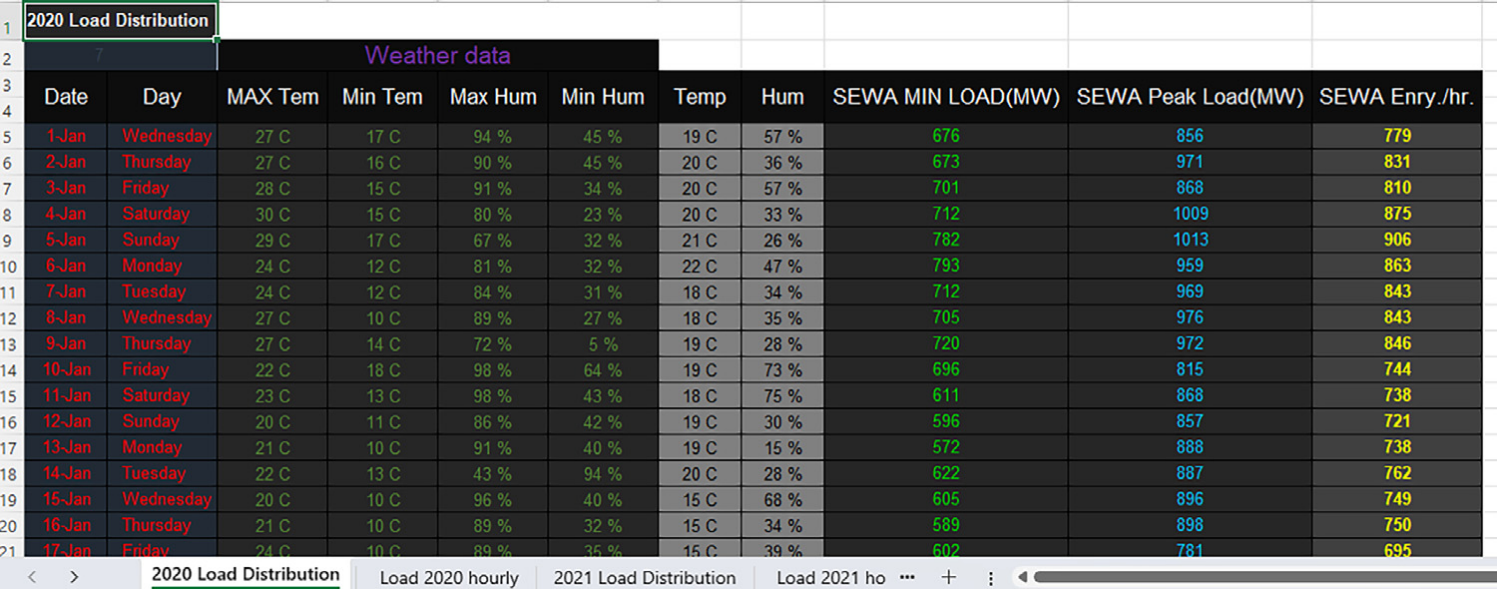
2020 Daily Load distribution considering temperature and humidity daily factors.

Excel File: SEWA_Daily_Demand_2020.xlsx: Differing slightly in structure, each row in this file corresponds to an individual day in 2020. Instead of hourly breakdowns, this sheet offers an aggregate or cumulative demand for each day taking in consideration the temperature and humidity daily factors. The following Fig. 2, depicts the load forecasting at daily basis considering the weather factors of temperature and humidity

**Fig. 3:**
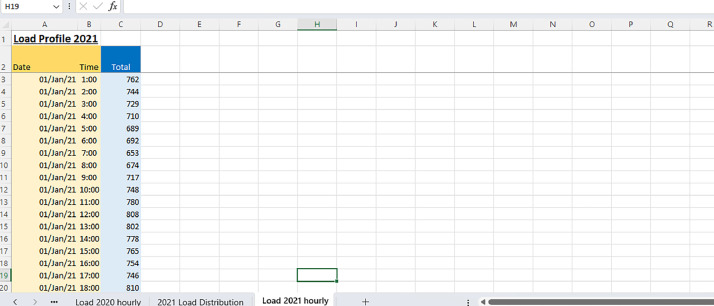
2021 Hourly collected data.

Excel File: Hourly_Demand_2021.xlsx: Similar in structure to its 2020 counterpart, this file enumerates the hourly electricity demand for every day of 2021. The following Fig. 3 represents the hourly collected data in each day of the 2021 year.

**Fig. 4:**
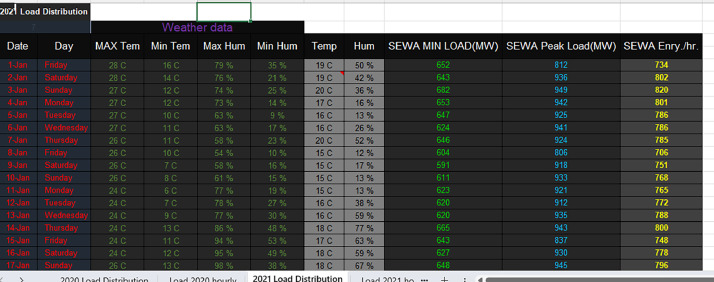
2021 Daily Load distribution considering temperature and humidity daily factors.

Excel File: SEWA_Daily_Demand_2021.xlsx: Mirroring the format of the 2020 version, this file gives the cumulative daily electricity demand figures for the entirety of 2021 considering temperature and humidity factors in particular day. The following Fig. 4, illustrates the daily load calculated demand based on 2021 weather factors of temperature and humidity daily.

## 3 Experimental Design and Methods

### 3.1 The different aspects of the importance of accurately predicting electricity demand

Electricity demand forecasting is of paramount importance in the evolving energy landscape. As the integration of renewable energy sources into the grid becomes more pronounced and the shift towards a more decentralized energy system gains momentum, the ability to accurately predict electricity demand is crucial.

Grid Management and Reliability: As grids become smarter and more interconnected, there's an increasing need for precise demand forecasts. Accurate forecasting ensures grid stability and prevents potential blackouts or brownouts. It also aids in effective load management during peak hours [1].

Integration of Renewables: With the surge in renewable energy adoption, notably wind and solar, there's inherent unpredictability in power generation. Accurate demand forecasting can offset this variability, ensuring a balance between demand and supply [2].

Optimizing Energy Storage: Energy storage solutions, especially batteries, play a pivotal role in maintaining grid balance. Knowing when the demand peaks will occur can help in optimizing battery charging and discharging cycles [3].

Pricing and Trading: In energy markets, prices fluctuate based on demand and supply. Accurate demand forecasting can be a competitive advantage for traders and can also help in setting dynamic pricing models for consumers [4].

Infrastructure Investment: For long-term planning, utilities need to invest in infrastructure. Accurate demand forecasting ensures that these investments are neither over nor under the actual requirements, saving costs and resources [5].

Supporting Policy Decisions: Policymakers can utilize demand forecasts to craft informed regulations and guidelines, especially in regions aiming for ambitious renewable energy targets or electrification programs [6].

The aforementioned utilities of electricity demand forecasting underscore its vital role in contemporary energy systems. It acts as a linchpin, ensuring efficiency, stability, and sustainability in the face of rapidly evolving energy demands and generation paradigms.

The following section describes how you acquired the data. Provide a complete description of the experimental design and methods.

### 3.2 Experimental design

The data acquisition for SEWA electricity forecasting was structured to capture temporal nuances, considering both hourly and daily fluctuations. Emphasis was placed on understanding the dynamic relationship between environmental conditions and electrical load metrics, which holds significant relevance for electricity demand forecasting.

### 3.3 Data acquisition methods

Hourly & Daily Data Collection:

Data was sourced for a two-year span (2020 & 2021) to capture both short-term and long-term trends.

Hourly data breakdown was executed to get power electricity demand metrics ranging from 0:00 to 24:00. Daily data representation followed, where each row symbolized the electricity demand values for that particular day or period. The following section is added in response to the reviewer comment:

Upon reviewing the “2020 Hourly Collected Data Sheet,” it does indeed appear that there are eight different measurements grouped together, with their sum shown in the ninth row. This grouping and summation of data may have been done to simplify the presentation of the information and provide an aggregate view of the eight measurements. This approach can make it easier to interpret the data, especially if the individual measurements are related or represent components of a larger variable.

The 2021 hourly data, on the other hand, are presented differently from the 2020 data. In the 2021 dataset, each measurement is provided individually for every hour without being grouped and summed in the same manner as the 2020 data. The difference in presentation is due to various factors, including changes in data collection and reporting practices, or it may reflect different data requirements or analysis needs for the two years.

Overall, the choice to group and sum certain measurements in the 2020 dataset and present the 2021 data differently is driven by specific data management or analytical considerations, making it easier to work with and analysing the data for the respective years.

### 3.4 Environmental metrics collection

For each hourly data point, associated environmental metrics such as temperature, humidity, wind speed, and solar irradiance were recorded.

The daily dataset integrated metrics such as MAX Temperature, MIN Temperature, MAX Humidity, etc.

### 3.5 SEWA load metrics integration

Alongside the environmental metrics, specific electrical load metrics like SEWA MIN LOAD(MW), SEWA Peak Load(MW), and SEWA Energy/hr were embedded in the data.

### 3.6 Lagged demand analysis

To understand the temporal dependencies better, the dataset also captured the demand value from the preceding hour.

### 3.7 Impact of Covid-19 pandemic

In analysing the loaded datasets for 2020 and 2021, inconsistent patterns in electricity load data were observed and linked to the effects of the COVID-19 pandemic lockdown. In 2020, a substantial reduction in electricity demand during the early months, particularly in March and April, coincided with the implementation of lockdowns and restrictions. This decline was evident in both general load profiles and specific SEWA load data. However, in 2021, the patterns shifted, revealing a return to normal or even increased electricity demand, indicating that the initial lockdown effects had largely dissipated. These findings underscore the transient nature of the pandemic's impact on electricity consumption and suggest the role of various factors, such as remote work practices and economic recovery, in shaping electricity demand during and after the lockdown period.

### 3.8 Instruments and software

Data Collection Instruments: Advanced electrical load meters were used to fetch real-time electrical load metrics. For environmental conditions, standard weather data acquisition systems, consistent with meteorological standards, were employed.

### 3.9 Software for data analysis

Excel: Primary tool used for initial data cleaning, organization, and preliminary analysis.

Python: Leveraged for advanced data processing tasks, especially for feature engineering. Libraries such as pandas and NumPy facilitated data manipulation, while matplotlib and seaborn enabled visual analysis.

### 3.10 Data processing

The raw data underwent rigorous cleaning to handle missing values, outliers, and any inconsistencies. Feature engineering was primarily executed using Python, where lagged variables, rolling averages, and other transformations were carried out to enhance the dataset's robustness.

## Limitations

While the dataset provides a comprehensive look into SEWA's electricity demand and associated environmental parameters over two years, several limitations are noteworthy:

Temporal limitations: The data only spans the years 2020 and 2021. As such, it may not capture long-term trends or anomalies in power consumption beyond this period.

Environmental data: The climatic conditions, though included, are limited to basic factors like temperature, humidity, and wind speed. Nuanced environmental metrics such as atmospheric pressure or pollutant levels are absent, potentially missing critical predictors for power demand.

Spatial limitations: The dataset represents SEWA's electric demand but may not be generalized to regions with distinct geographic, climatic, or socio-economic characteristics.

Data granularity: Although hourly breakdowns are available, minute-to-minute variations, which could be instrumental in studying short-term spikes or drops, are missing.

Lagged demand representation: Only one-hour lag data is provided. Inclusion of multiple lag hours might have enriched the dataset further for time-series analysis.

Overall, while the dataset serves as a valuable resource for studying electricity demand in SEWA's context during 2020-2021, these limitations should be considered when using the data for research or forecasting purposes.

## Ethics Statement

The authors have read and follow the ethical requirements for publication in Data in Brief and confirm that the current work does not involve human subjects, animal experiments, or any data collected from social media platforms.

## CRediT authorship contribution statement

**M.S. Jawad:** Conceptualization, Methodology, Software, Writing – original draft, Investigation. **Chitra Dhawale:** Supervision, Validation, Writing – review & editing. **Abdel Rahman Al Ali:** Validation, Writing – review & editing. **Azizul Azhar Bin Ramli:** Validation, Writing – review & editing.

## Data Availability

SEWA Electricity Deman Forecasting – 2020 and 2021 (Original data) (Mendeley Data).
